# Survival-Associated Alternative Splicing Events in Pan-Renal Cell Carcinoma

**DOI:** 10.3389/fonc.2019.01317

**Published:** 2019-11-27

**Authors:** Keren Jia, Yingcheng Wu, Jing Huang, Huiqun Wu

**Affiliations:** ^1^Medical School of Nantong University, Nantong, China; ^2^School of Pharmacy, Nanjing University of Chinese Medicine, Nanjing, China; ^3^Department of Medical Informatics, Medical School of Nantong University, Nantong, China

**Keywords:** alternative splicing, splicing factor, renal cell carcinoma, prognosis, bioinformatics

## Abstract

Alternative splicing is an important modification process for the genome to generate mature mRNA by transcription, which has been found associated with survival in some tumors. However, systematic analysis of AS events in pan-renal cell carcinoma at the genome-wide level has been seldom conducted yet. In the current study, Upset plot and Venn plot were utilized to present the distribution characteristics of AS events. Those SREs were screened out with multivariate COX regression analyses, and functional enrichment analysis was performed to figure out potential pathways. ROC model was conducted to compare the efficiency of those potential SREs. A total of 2,169, 1,671, and 1,414 SREs were found in renal clear cell carcinoma (KIRC), renal chromophobe cell carcinoma (KICH), and renal papillary cell carcinoma (KIRP), respectively. Functional enrichment analysis results suggested possible mechanism such as changes in the branched-chain amino acid catabolic process due to SREs might play a key role in KIRC. The binary logistic regression equation based on the SREs had a good performance in each model compared to the single factor. The 5 year survival model presented that the AUC of the predicted probabilities in KIRC, KICH, and KIRP were 0.754, 1 and 0.841, and in the diagnostic model were 0.988, 0.970, and 0.999, respectively. Some AS types that were significantly different in pan-RCC and paracancerous tissues have also been discovered to play a role in carcinoma screening. To sum up, alternative splicing events significantly interfere with the prognosis of patients with pan-RCC and are capable as biomarkers for prognosis.

## Introduction

Alternative splicing (AS) refers to the fact that a pre-mRNA produces different mRNA splicing isoforms at different splice sites through different splicing methods, which is essential for the regulation of gene expression and the production of protein diversity (1). AS is considered to be the root cause of eukaryotes with significantly fewer genes than protein species. Under normal conditions, AS events is precisely regulated, which contributes to physiological functions, such as the immune system (2). Abnormal AS events will affect tumor cell differentiation, apoptosis, invasion, and metastasis by affecting gene expression products (3). Even in the absence of genetic mutations, some cancer-associated AS events may lead to carcinogenesis, which may be associated with mutations in the intron splice sites of tumor suppressor genes and become potential therapeutic targets (4). Hence, the study of AS on cancer has becomes a hot area.

Pan-renal cell carcinoma (pan-RCC) includes renal clear cell carcinoma (KIRC), renal chromophobe cell carcinoma (KICH), and renal papillary cell carcinoma (KIRP), accounting for 80–90% of renal malignancies (5). Other rare cancers (including duct carcinoma, renal medullary carcinoma, and urothelial carcinomas) with low incidence also occur in the kidneys (6, 7). The classification of RCC based on pathology model is widely accepted, but studies have shown that morphological parameters cannot be used as an effective indicator for prognosis (8). Some researchers have classified RCC into nine major types based on multidimensional and comprehensive molecular characterization (9). In addition, gene mutations, gene expression profiles, and inflammatory markers have also attracted attention in the development and prognosis of RCC (10–12).

The research evidence in recent years partly brings to lights the ways in which AS affects RCC. PTBP1 plays a tumorigenic role in KIRC by mediating PKM2 AS, and it may be a potential prognostic marker as well as a promising molecular target for the treatment of KIRC (13). Epithelial splicing regulatory protein 2 (ESRP2) is one of the key regulators of AS in epithelial cells, expressed in KIRC, whereas ESRP1 is downregulated in most KIRC patients (14). Interpretation of splicing factors (SFs) expression in KIRC may result in selective splicing damage of genes regulating tumor growth, and this approach contributes to the carcinogenesis process (15). These studies focus on KIRC, demonstrating the decisive position of AS events in influencing the production of RCC.

Considering AS events could be a diagnostic and prognostic marker, even be a new classification basis for pan-RCC, the investigations on AS events in pan-RCC is imperative. Based on RNA sequencing data, we systematically analyzed AS events in pan-RCC and paracancerous tissues, as well as identified SREs in the three subtypes of pan-RCC. Furthermore, the potential of these SREs in the diagnosis of RCC was validated. Mapping regulatory networks of genes in SREs for KIRC, KICH, and KIRP sharpens our insight into understanding the specific pathways by which AS acts on RCC.

## Materials and Methods

### Data Acquisition and Preprocessing

The TCGA SpliceSeq database systematically identified mRNA splicing events in 33 tumors (total number of samples >10,000) in the TCGA database, each tumor data including high-throughput sequencing data, AS events, and partial clinical information for cancerous and paracancerous tissues (16). Since the clinical data in TCGA SpliceSeq is not comprehensive enough, all clinical data was downloaded from the TCGA database for more detailed analysis. SpliceSeq, a Java application that more intuitively demonstrates the AS pattern in high-throughput sequencing data by calculating the Percent-Spliced-In (PSI) value for each event (17). PSI values are used to quantify each AS event, making it possible to analyze AS events using biometric methods. TCGA SpliceSeq classifies AS events into seven types: Exon Skip (ES), Retained Intron (RI), Mutually Exclusive Exons (ME), Alternate Donor site (AD), Alternate Acceptor site (AA), Alternate Promoter (AP), and Alternate Terminator (AT). The PSI values for seven types of AS events in the three tumors contained in pan-RCC were downloaded from TCGA SplicSeq. We removed the events that contained the vacancy values to make the results more reliable. Finally, data for KIRC were obtained from 605 samples (533 cancer tissues and 72 adjacent cancer tissues). Data for KICH were obtained from 91 samples (66 cancer tissues and 25 adjacent cancer tissues). Data for KIRP were obtained from 322 samples (290 cancer tissues and 32 adjacent cancer tissues). Cancer tissue samples and some paracancerous tissue samples from different patients were obtained. Each sample could be matched to corresponding patient to acquire their clinical information.

### Multivariable Survival Analysis

A total of 516 patients with KIRC, 64 patients with KICH, and 276 patients with KIRP were included in the survival analysis. Patients with a total survival of <30 days or >5,000 days in clinical data were omitted. Cox's proportional hazards regression model was used to calculate the relationship between PSI values and overall survival (OS) in patients with cancer, the results of which includes the coef value, 95% confidence intervals, and *P*-values. Only AS events with a *P* < 0.05 were considered to be potentially relevant to survival. The coef value is a key parameter that reflects the impact and direction of the event on prognosis. A positive coef value would increase the risk of death, while a negative value would reduce the risk of death. The magnitude of the value is related to the degree of impact. Life activities are the combined result of a variety of AS events. The PSI value of some SREs was multiplied by the coef value to obtain a weighted PSI value for each patient, which was used to analyze the correlation of multifactors with survival. A more objective reflection of the impact of AS events on patient survival will be obtained in this way. We performed independent factor survival analysis for events incorporating multivariate analysis as well. The patients were isolated into two groups by the median of the single event PSI value and the multi-event weighted PSI values for all patients. The Kaplan-Meier (K-M) survival analysis was used to see if there was a significant difference in prognosis between the two groups. This algorithm is implemented by survival and survminer, two R language packages, which can be downloaded and installed from Bioconductor (18). Outcomes with a *P* < 0.05 were considered to be statistically different.

### Upset Plot and Venn Plot

Upset plot is the inheritance and development of venn plot, which can more intuitively display the intersection of multiple sets (usually ≥5). When the number of sets is <5, the venn plot showed better readability. Upset plots presented the intersection of seven types of all AS events and related genes or only survival related events and genes in pan-RCC. The venn plot was drawn only for cross-tumor analysis to compare the distribution of AS events and related genes in pan-RCC (19).

### Protein-Protein Interaction (PPI) Network and Enrichment Analysis

In order to gain insight into how genes involved in potential SREs perform mutual regulation in pan-RCC, these genes were submitted to the STRING database (www.string-db.org/) for constructing a PPI network. The threshold is set to 0.9, helping us get more reliable data. In the PPI network, a gene with higher degree is considered to be hub gene, indicating its central position in the regulatory network. They were submitted to the GO and KEGG database for enrichment analysis as well, figuring out the functions and pathways involved in SREs (20, 21).

### Statistical Analysis

The receiver operating characteristic curve (ROC) combines sensitivity and specificity in a graphical manner that accurately reflects the relationship between specificity and sensitivity of an analytical method, proven to be a reliable method for testing the diagnostic value of an indicator for a disease (22–24). In each tumor, the PSI values of the 10 most significant events and the weighted PSI value obtained by weighting these events were used for ROC analysis to comprehensively compare the power of predicting outcomes in 5 year survival models. Considering that some factors may improve or worsen the prognosis of the disease, but work as a criterion for diagnosing the disease, we explored the ability of the PSI values of the 10 events with significant prognosis and the weighted PSI value in terms of selecting tumor tissues from all tissues. We fit the binary logistic regression equation using the PSI values of the 10 most significant events and compare the predicted probabilities with the weighted PSI values, with the help of the ROC curve. In addition, the study examined whether PSI values for each type of AS event differed between cancerous and paracancerous tissues. Whether these indicators are effectively classified for cancer tissues and adjacent tissues is also tested. The calculation of binary logistic regression equation and ROC analysis are realized by SPSS19.0 software (SPSS Inc., Chicago, IL) (25, 26).

### The Regulatory Network Containing Splicing Factors (SFs)

A total of 68 SFs were found to be involved in the regulation of AS events in pan-RCC, which were available from the SpliceAid 2 (www.introni.it/spliceaid.html) database (27). TCGA provided a level three gene expression profile of KIRC, KICH, and KIRP. The original read counts were normalized to eliminate differences in the total amount of data, gene length, and number of genes, ensuring the reliability of the results. Univariate COX regression analysis was used to mine survival-related SFs. The Pearson correlation coefficient was used to measure the regulatory relationship between SFs and AS events.

## Results

### Distribution of AS Events as Well as Related Genes in Pan-RCC

In KICH, there were a total of 10,226 genes involved in 29,722 AS events, which were identified AS-related genes (ASRGs). We found 2,446 genes in 3,263 AAs, 2,141 genes in 2,759 ADs, 3,489 genes in 3,489 APs, 3,642 genes in 3,642 ATs, 6,542 genes in 13,728 ESs, 155 genes in 157 MEs, and 1,839 genes in 2,684 RIs. In KIRC, there are a total of 10,567 genes involved in 30,979 AS events. We found 2,562 genes in 3,416 AAs, 2,192 genes in 2,813 ADs, 3,620 genes in 3,620 APs, 3,729 genes in 3,729 ATs, and 6,840 genes in 14,451 ESs, 170 genes in 173 MEs, and 1,902 genes in 2,777 RIs. In KIRP, there are a total of 9,988 genes involved in 27,820 AS events. We found 2,285 genes in 3,023 AAs, 1,974 genes in 2,531 ADs, 3,201 genes in 3,201 APs, 3,661 genes in 3,661 ATs, and 6,221 genes in 12,634 ESs, 130 genes in 130 MEs, and 1,802 genes in 2,640 RIs.

**Figure 1:**
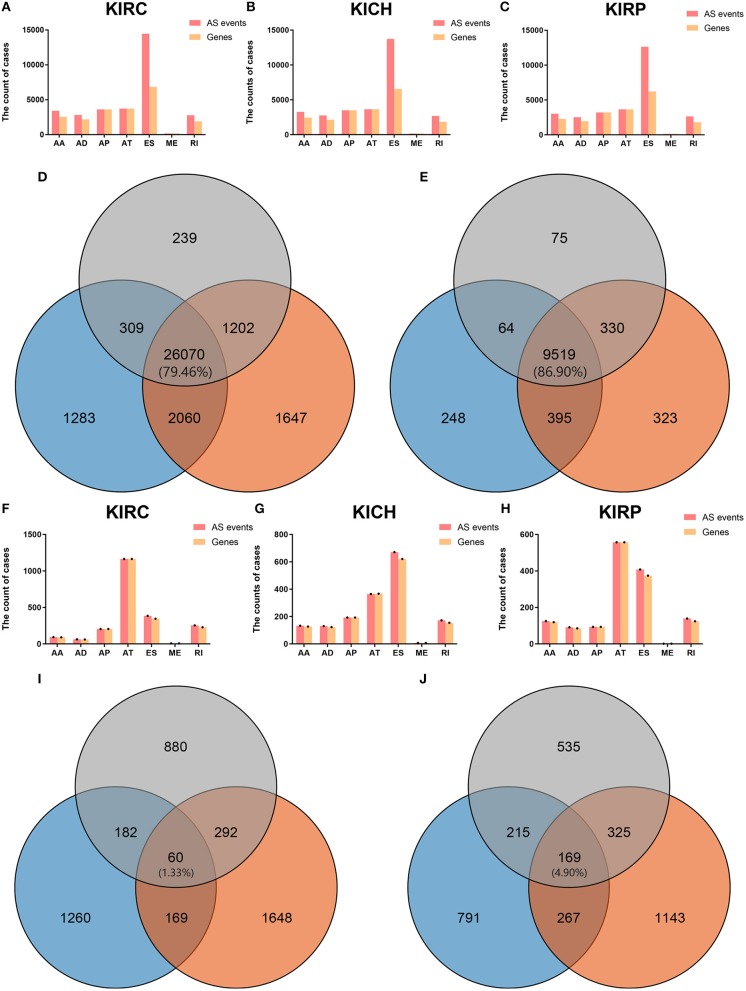
The AS events, SREs and relevant genes in RCC. **(A–C)** The height of the pink strip represented the number of AS events. The height of the orange band represented the number of relevant genes. **(D)** The orange, blue, and gray areas represent the events in KIRC, KICH, and KIRP, respectively. The percentage value in the middle represented the ratio of the AS events co-existing in the three sets to all AS events. **(E)** The orange, blue, and gray areas represent the genes in KIRC, KICH, and KIRP, respectively. The percentage value in the middle represented the ratio of the ASRGs co-existing in the three sets to all ASRGs. **(F–H)** The height of the pink strip represented the number of SREs. The height of the orange strip represented the number of SRGs. Because the number of MEs and ME related genes was too small; black dots on the bar graph indicated their existence. **(I)** The orange, blue, and gray areas represent the SREs in KIRC, KICH, and KIRP, respectively. The percentage value in the middle represented the ratio of the SREs co-existing in the three sets to all SREs. **(J)** The orange, blue, and gray areas represent the SRGs in KIRC, KICH, and KIRP, respectively. The percentage value in the middle represented the ratio of the SRGs co-existing in the three sets to all SRGs.

**Figure 2:**
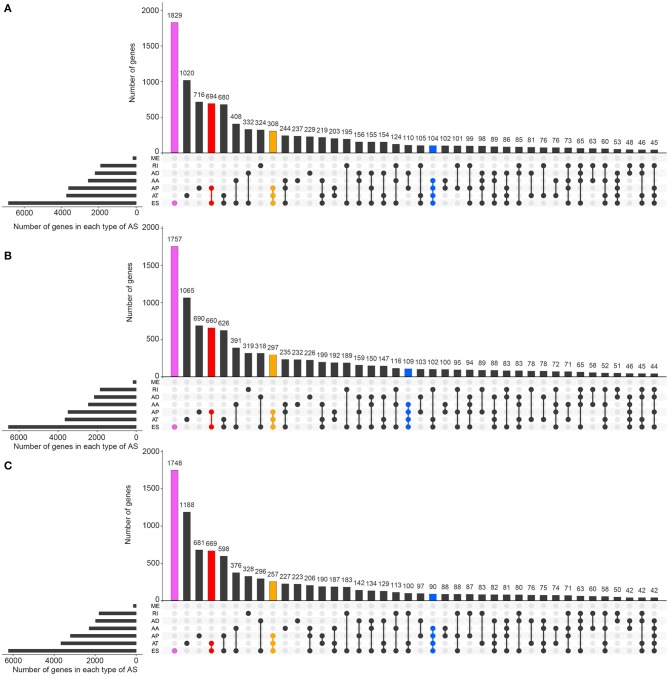
The distributions of ASEs and ASRGs in each kind of RCC. The Upset plot shows the distribution of ASRGs and ASEs in the pan-RCC. The bar chart above represents the number of genes contained in each type of group. The bar chart at the bottom left represents the number of events included in each type of AS events. The dotted line at the bottom right shows the types of events contained in the group. Pink indicates the group with the largest number of ASRGs in the groups containing one event type. Red indicates the group with the largest number of ASRGs in the groups containing two event types. Yellow indicates the group with the largest number of ASRGs in the groups containing three event types. Blue indicates the group with the largest number of ASRGs in the groups containing four event types. **(A)** KIRC; **(B)** KICH; and **(C)** KIRP.

Figures 1A–C visually presented the contrast between AS events and ASRGs in pan-RCC. Interestingly, ADs and ATs are identical in number to related genes, which can be observed in all subtypes of pan-RCC. In each type of RCC, ESs are the most AS events, and MEs are the fewest AS events. The common gene distribution of seven types of AS events was shown in Figure 2. In KIRC and KICH, the AP&ES gene group contained 694 and 660 genes, respectively, which was the group with the largest number of genes in all groups with genes involved in two types of events. However, in KIRP, the group with the largest number of genes involved in two types of events is the AT&ES gene group, containing 669 genes. In each subtype of pan-RCC, the groups with the largest number of genes were involved in three or four types of events are AP&AT&ES and AA&AP&AT&ES gene group, respectively. 79.46% of AS events and 86.90% of ASRGs were present in all subtypes of pan-RCC, more details could be found in Figures 1D,E. Overall, pan-RCC has the similar ratio of AS events/ASRGs and distribution characteristics of AS events and ASRGs, suggesting that they may have associated pathological features.

### Splicing Feature of SREs and SRGs in Pan-RCC

Biological processes are the result of interactions between multiple AS events. The effects of individual factors on outcomes can be quantified by multivariate survival analysis. In KIRC, 1,904 candidate genes (SRGs) are present in 2,169 SREs, including 91 genes in 93 AAs, 60 genes in 63 ADs, and 204 genes in 204 APs, 1,164 genes in 1,164 ATs, 345 genes in 384 ESs, 7 genes in 7 MEs, and 228 genes in 254 RIs (Figure 1F). For KICH, 1,442 SRGs are present in 1,671 SREs, including 127 genes in 133 AAs, 123 genes in 130 ADs, and 193 genes in 193 APs, 365 genes in 365 ATs, 621 genes were in 671 ESs, 7 genes in 7 MEs, and 154 genes in 172 RIs (Figure 1G). In KIRP, 1,244 SRGs were observed to involve in 1,414 SREs, including 119 genes in 125 AAs, 85 genes in 91 ADs, and 93 genes in 93 APs, 557 genes in 557 ATs, 374 genes in 408 ESs, 1 gene in 1 MEs, and 124 genes in 139 RIs (Figure 1H). The detailed information about SREs was presented in Supplementary Table 1.

**Figure 3:**
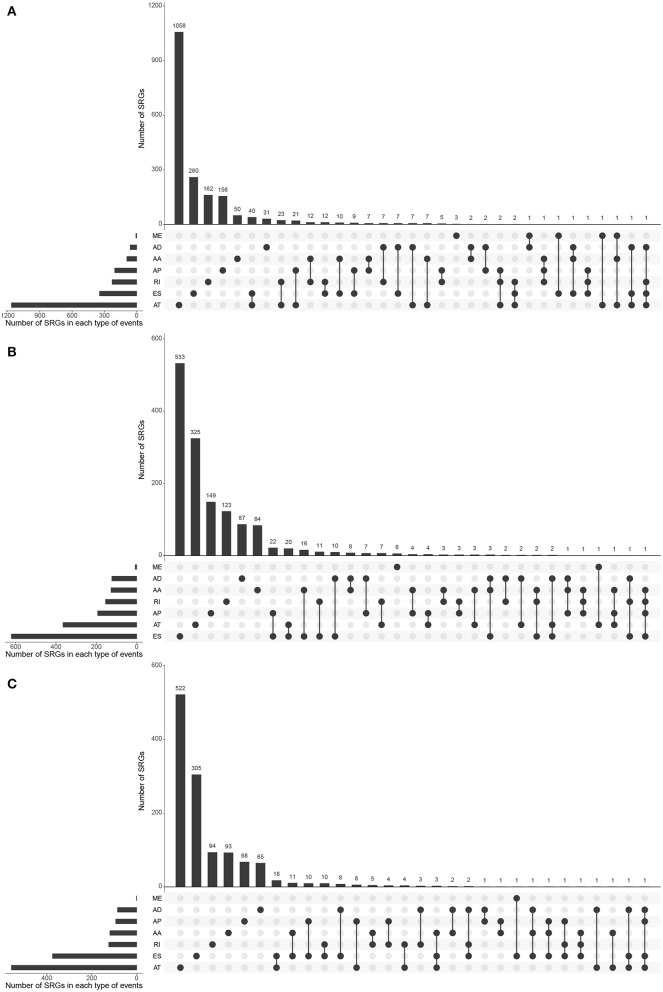
The distributions of SREs and SRGs in each kind of RCC. The strip at the bottom left shows the number of SREs included in each AS type. The dot and line at the bottom right represent the subsets of AS events. The AS types corresponding to dots were contained in the subtype. The number of relevant genes in each subset is represented in the histogram, which is the upper part of the whole plot. **(A)** KIRC; **(B)** KICH; and **(C)** KIRP.

Most of the genes affecting prognosis occur only one AS event. ATs are the main AS types affecting the prognosis of patients with KIRC and KIRP, while ESs are the main one in KICH. In pan-RCC, only a very small number of MEs contribute to prognosis (Figure 3). Among all SRGs, 4.90% of SRGs were present in three types of renal cell carcinomas. For SREs, this ratio is 1.33%, which is less than one-third that of SRGs (Figures 1I,J), suggesting that different pathological processes alter the prognosis of the subtype of pan-RCC, which are more dependent on SREs than SRGs.

### Prognostic Models Based on SREs

**Figure 4:**
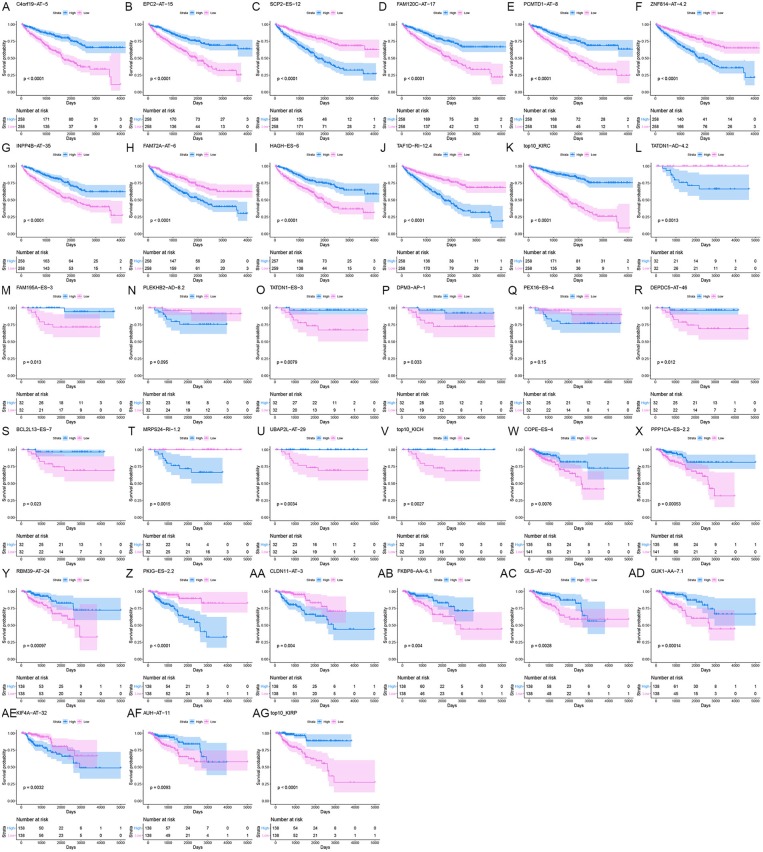
The survival curve for different prognostic factors. In the upper graph, the blue curve represented the group with high PSI values; the pink curve represented the group with low PSI values. The table below showed the number of people at risk that change over time. **(A)** Survival curve based on the PSI value of C4orf19_AT_5 in KIRC. **(B)** Survival curve based on the PSI value of EPC2_AT_15 in KIRC. **(C)** Survival curve based on the PSI value of SCP2_ES_12 in KIRC. **(D)** Survival curve based on the PSI value of FAM120C_AT_17 in KIRC. **(E)** Survival curve based on the PSI value of PCMTD1_AT_8 in KIRC. **(F)** Survival curve based on the PSI value of ZNF814_AT_4.2 in KIRC. **(G)** Survival curve based on the PSI value of INPP4B_AT_35 in KIRC. **(H)** Survival curve based on the PSI value of FAM72A_AT_6 in KIRC. **(I)** Survival curve based on the PSI value of HAGH_ES_6 in KIRC. **(J)** Survival curve based on the PSI value of TAF1D_RI_12.4 in KIRC. **(K)** Survival curve based on the weighted PSI value of KIRC. **(L)** Survival curve based on the PSI value of TATDN1_AD_4.2 in KICH. **(M)** Survival curve based on the PSI value of FAM195A_ES_3 in KICH. **(N)** Survival curve based on the PSI value of PLEKHB2_AD_8.2 in KICH. **(O)** Survival curve based on the PSI value of TATDN1_ES_3 in KICH. **(P)** Survival curve based on the PSI value of DPM3_AP_1 in KICH. **(Q)** Survival curve based on the PSI value of PEX16_ES_4 in KICH. **(R)** Survival curve based on the PSI value of DEPDC5_AT_46 in KICH. **(S)** Survival curve based on the PSI value of BCL2L13_ES_7 in KICH. **(T)** Survival curve based on the PSI value of MRPS24_RI_1.2 in KICH. **(U)** Survival curve based on the PSI value of UBAP2L_AT_29 in KICH. **(V)** Survival curve based on the weighted PSI value of KICH. **(W)** Survival curve based on the PSI value of COPE_ES_4 in KIRP. **(X)** Survival curve based on the PSI value of PPP1CA_ES_2.2 in KIRP. **(Y)** Survival curve based on the PSI value of RBM39_AT_24 in KIRP. **(Z)** Survival curve based on the PSI value of PKIG_ES_2.2 in KIRP. **(AA)** Survival curve based on the PSI value of CLDN11_AT_3 in KIRP. **(AB)** Survival curve based on the PSI value of FKBP8_AA_6.1 in KIRP. **(AC)** Survival curve based on the PSI value of GLS_AT_20 in KIRP. **(AD)** Survival curve based on the PSI value of GUK1_AA_7.1 in KIRP. **(AE)** Survival curve based on the PSI value of KIF4A_AT_32 in KIRP. **(AF)** Survival curve based on the PSI value of AUH_AT_11 in KIRP. **(AG)** Survival curve based on the weighted PSI value of KIRP.

**Figure 5:**
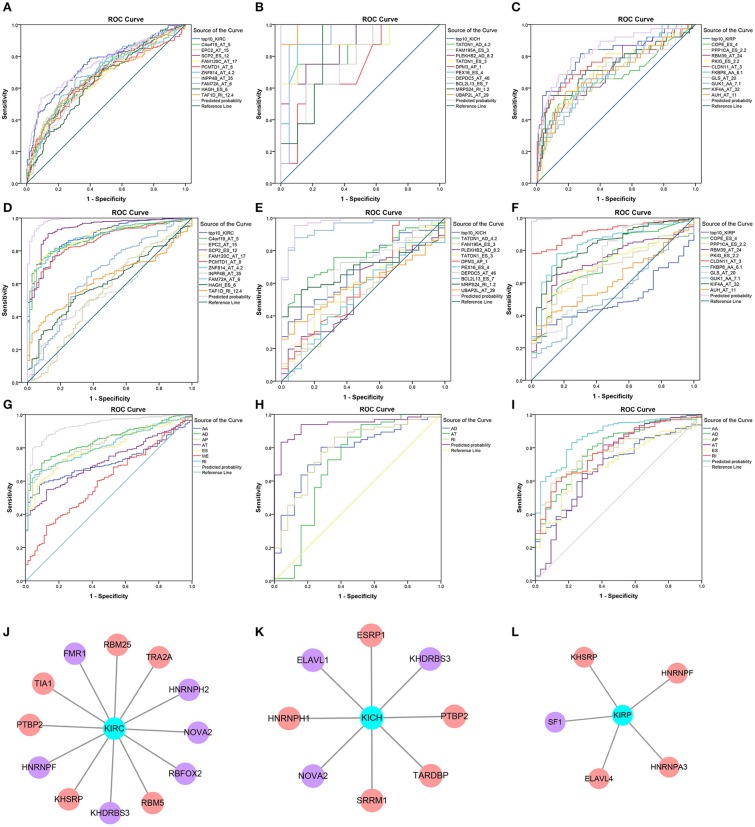
Survival-related SFs in pan-RCC and the ROC curve of three model. **(A–I)** The ROC curves of 5 year survival model in KIRC, KICH, and KIRP was shown in **(A–C)**. The ROC curves of the diagnostic model using the PSI value of SREs in KIRC, KICH, and KIRP was shown in **(D–F)**. The ROC curves of diagnostic model using the weighted PSI value of each AS type in KIRC, KICH, and KIRP were shown in **(G–I)**. The different color of the lines represented different predictors, the detailed information of which presented in the illustration on the right of figures. The X axis was a (1-Specificity) value. The y-axis was the sensitivity value. **(J–L)** The survival-related SFs in KIRC, KICH, and KIRP, respectively. The red nodes represented the SFs that are positively related to survival. The purple nodes represented the SFs that are negatively related to survival. The cyan node represents the type of RCC.

Information on the 10 most significant SREs in pan-RCC was shown in Table 1. All entries in it have been selected with *P* < 0.05. We established two prognostic models based on the most significant SREs. The K-M survival curve presented the trend of survival over time for univariate and multivariate survival analyses (Figure 4). Univariate survival analysis usually showed the impact of PSI values on survival. However, it could be clearly seen that when multi-factor weighted PSI values were used for grouping, the difference in survival between the high expression group and the ground expression group was more pronounced (Figures 4K,V,AG). The ROC curve compared the 5 year survival outcomes of patients with different factors (Figures 5A–C), and more details were recorded in Table 2. The AUC value was regarded as an indicator for judging the prediction effect. In pan-RCC, the weighted PSI values always exhibited better or the same predictive effect than any single SREs. When using SREs to fit a binary logistic regression equation, the AUC values of predicted probability were better or equal than that of the weighted PSI values in most AS events.

**Table 1:** The information of top10 survival related events.

**Type**	**Events**	**ID**	**Coef**	**95% CI lower**	**95% CI upper**	***P*-value**
KIRC						
	C4orf19_AT_5	69001	−2.873	−3.520	−2.227	<0.05
	EPC2_AT_15	55538	−6.284	−7.772	−4.796	<0.05
	SCP2_ES_12	3045	2.837	2.116	3.557	<0.05
	FAM120C_AT_17	89238	−3.433	−4.306	−2.560	<0.05
	PCMTD1_AT_8	83807	−14.851	−18.695	−11.008	<0.05
	ZNF814_AT_4.2	52355	8.437	6.236	10.638	<0.05
	INPP4B_AT_35	70691	−7.025	−8.867	−5.184	<0.05
	FAM72A_AT_6	9578	3.849	2.817	4.881	<0.05
	HAGH_ES_6	33146	−27.773	−35.307	−20.240	<0.05
	TAF1D_RI_12.4	18313	3.940	2.871	5.008	<0.05
KICH						
	TATDN1_AD_4.2	85085	19.167	10.734	27.600	<0.05
	FAM195A_ES_3	32927	−21.988	−32.280	−11.697	<0.05
	PLEKHB2_AD_8.2	55376	29.819	15.557	44.082	<0.05
	TATDN1_ES_3	85090	−33.292	−49.233	−17.352	<0.05
	DPM3_AP_1	7946	−89.941	−133.516	−46.366	<0.05
	PEX16_ES_4	15523	84.717	43.551	125.883	<0.05
	DEPDC5_AT_46	61896	−98.093	−146.147	−50.038	<0.05
	BCL2L13_ES_7	96058	−31.186	−46.554	−15.819	<0.05
	MRPS24_RI_1.2	79352	42.536	21.439	63.634	<0.05
	UBAP2L_AT_29	7814	−25.624	−38.488	−12.760	<0.05
KIRP						
	COPE_ES_4	48520	−167.002	−214.158	−119.846	<0.05
	PPP1CA_ES_2.2	17184	−68.743	−88.308	−49.178	<0.05
	RBM39_AT_24	59235	−77.423	−99.691	−55.155	<0.05
	PKIG_ES_2.2	59481	7.228	5.056	9.400	<0.05
	CLDN11_AT_3	67616	3.696	2.574	4.818	<0.05
	FKBP8_AA_6.1	48446	−16.366	−21.358	−11.373	<0.05
	GLS_AT_20	56589	−5.232	−6.861	−3.603	<0.05
	GUK1_AA_7.1	10188	−45.194	−59.292	−31.096	<0.05
	KIF4A_AT_32	89373	4.438	3.045	5.832	<0.05
	AUH_AT_11	86823	−11.438	−15.095	−7.780	<0.05

*Events are identified as symbol_splice type_exons. ID is the unique number used by The TCGA SpliceSeq database to represent each AS event in each kind of tumor. Coef values are used to represent the quantitative relationship between variables and results. Absolute values represent correlation strength. Positive numbers represent positive correlations. Negative numbers represent negative correlations; CI, confidence interval*.

**Table 2:** ROC curve results of survival related factors in predicting 5 year survival.

**Cancer type**	**Events**	**Cut-off**	**Sensitivity**	**Specificity**	**AUC**	**95% CI lower**	**95% CI upper**	***P*-value**
KIRC								
	Top10_KIRC	−53.062	0.748	0.641	0.748	0.696	0.800	<0.05
	C4orf19_AT_5	0.759	0.646	0.627	0.681	0.625	0.737	<0.05
	EPC2_AT_15	0.864	0.490	0.809	0.671	0.614	0.728	<0.05
	SCP2_ES_12	0.412	0.585	0.714	0.682	0.626	0.738	<0.05
	FAM120C_AT_17	0.530	0.442	0.841	0.666	0.609	0.723	<0.05
	PCMTD1_AT_8	0.963	0.578	0.695	0.649	0.589	0.708	<0.05
	ZNF814_AT_4.2	0.194	0.646	0.664	0.673	0.615	0.730	<0.05
	INPP4B_AT_35	0.943	0.701	0.573	0.669	0.612	0.725	<0.05
	FAM72A_AT_6	0.420	0.667	0.686	0.702	0.647	0.756	<0.05
	HAGH_ES_6	0.986	0.673	0.573	0.644	0.587	0.701	<0.05
	TAF1D_RI_12.4	0.271	0.565	0.732	0.666	0.608	0.724	<0.05
	Predicted probability	0.520	0.537	0.909	0.754	0.701	0.806	<0.05
KICH								
	Top10_KICH	−248.082	1.000	1.000	1.000	1.000	1.000	<0.05
	TATDN1_AD_4.2	0.268	0.750	0.895	0.862	0.709	1.000	<0.05
	FAM195A_ES_3	0.615	1.000	1.000	1.000	1.000	1.000	<0.05
	PLEKHB2_AD_8.2	0.170	0.750	0.842	0.868	0.722	1.000	<0.05
	TATDN1_ES_3	0.918	0.875	0.895	0.888	0.725	1.000	<0.05
	DPM3_AP_1	0.972	0.625	0.842	0.730	0.524	0.936	0.063
	PEX16_ES_4	0.024	0.875	0.895	0.931	0.837	1.000	<0.05
	DEPDC5_AT_46	0.978	0.625	0.842	0.763	0.576	0.950	<0.05
	BCL2L13_ES_7	0.822	0.875	0.947	0.921	0.810	1.000	<0.05
	MRPS24_RI_1.2	0.104	1.000	0.737	0.836	0.684	0.987	<0.05
	UBAP2L_AT_29	0.392	1.000	0.895	0.987	0.953	1.000	<0.05
	Predicted probability	0.500	1.000	1.000	1.000	1.000	1.000	<0.05
KIRP								
	Top10_KIRP	−375.499	0.816	0.724	0.817	0.734	0.901	<0.05
	COPE_ES_4	0.992	0.500	0.859	0.671	0.560	0.782	<0.05
	PPP1CA_ES_2.2	0.979	0.474	0.870	0.683	0.580	0.787	<0.05
	RBM39_AT_24	0.975	0.447	0.886	0.706	0.612	0.801	<0.05
	PKIG_ES_2.2	0.181	0.816	0.562	0.716	0.622	0.810	<0.05
	CLDN11_AT_3	0.189	0.684	0.773	0.753	0.650	0.856	<0.05
	FKBP8_AA_6.1	0.690	0.579	0.751	0.704	0.611	0.797	<0.05
	GLS_AT_20	0.734	0.658	0.708	0.688	0.584	0.792	<0.05
	GUK1_AA_7.1	0.962	0.658	0.686	0.702	0.610	0.794	<0.05
	KIF4A_AT_32	0.641	0.553	0.924	0.730	0.628	0.832	<0.05
	AUH_AT_11	0.876	0.579	0.816	0.698	0.598	0.798	<0.05
	Predicted probability	0.143	0.789	0.784	0.841	0.768	0.915	<0.05

*Events are identified as symbol_splice type_exons. Top10_KIRC means the weighted PSI value of the top10 significant SREs in KIRC. Top10_KICH means the weighted PSI value of the top10 significant SREs in KICH. Top10_KIRP means the weighted PSI value of the top10 significant SREs in KIRP.AUC, area under the ROC curve; CI, confidence interval*.

### Hub Genes in PPI Network

Hub genes are selected based on the number of genes connected. In KIRC, hub genes with higher degrees are: RPL9 (degree = 47), RPL27A (degree = 47), RPL26 (degree = 46), RPS15A (degree = 46), RPL17 (degree = 46), RPL15 (degree = 46), RPS9 (degree = 46), RPS20 (degree = 45), RPS3A (degree = 45), RPS6 (degree = 45), RPS25 (degree = 45), RPS5 (degree = 45), RPL35 (degree = 45). In KICH, 8 hub genes are RPS2 (degree = 16), RPL8 (degree = 16), RPL17 (degree = 16), RPS9 (degree = 16), RPS20 (degree = 15), RPS16 (degree = 15), RPL27A (degree = 15), RPL10 (degree = 15). Hub genes in KIRP are RPS20 (degree = 26), RPS15A (degree = 26), RPS29 (degree = 25), RPL23A (degree = 25), RPS3A (degree = 25), RPS15 (degree = 25), RPS19 (degree = 25), RPS7 (degree = 25). The interaction of all genes was shown in Supplementary Figure 1, with the color of nodes representing different degrees.

### Function and Pathway Enrichment Analysis

Based on all SRGs, we have identified 360 GO terms and eight KEGG terms in KIRC, 250 GO terms and two KEGG terms in KICH, 180 GO terms and eight KEGG terms in KIRP. Considering the large number of entries in the results, we present the 10 terms with the highest proportion of related gene genes and the 10 terms with the most genes involved in Tables 3, 4. From the number of participating genes, most of the SRGs in pan-RCC are involved in intracellular, intracellular organelle, cytoplasm, intracellular membrane-bounded organelle, cytoplasmic part, intracellular organelle part. From the perspective of functionally related genes, SRGs in KIRC were significantly involved in the branched-chain amino acid catabolic process, branched-chain amino acid metabolic process, cytosolic small ribosomal subunit, cotranslational protein targeting to membrane, SRP-dependent cotranslational protein targeting to membrane, protein targeting to ER, establishment of protein localization to endoplasmic reticulum, cytosolic ribosome, mitochondrial electron transport, NADH to ubiquinone, protein localization to endoplasmic reticulum. SRGs in KICH were significantly involved in protein localization to phagophore assembly site, phosphatidylinositol-3-phosphate binding, Golgi to plasma membrane protein transport, phosphatidylinositol-3,5-bisphosphate binding, Autophagy, peroxisome organization, establishment of protein localization to plasma membrane, nucleobase-containing small molecule interconversion, pre-mRNA binding, Golgi to plasma membrane transport. SRGs in KIRP were significantly involved in NADH dehydrogenase (quinone) activity, NADH dehydrogenase (ubiquinone) activity, NADH dehydrogenase complex, respiratory chain complex I, mitochondrial respiratory chain complex I, mitochondrial respiratory chain complex I biogenesis, NADH dehydrogenase complex assembly, mitochondrial respiratory chain complex I assembly, NADH dehydrogenase activity, mitochondrial proton-transporting ATP synthase complex. This result indicates that the significantly affected cell functions in pan-RCC are diverse, which may be responsible for pathological differences.

**Table 3:** The information of GO terms with the highest ratio.

	**ID**	**Term**	**Source**	***P*-value**	**% associated genes**
KIRC					
	GO:0009083	Branched-chain amino acid catabolic process	GO biological process	<0.01	52.17
	GO:0009081	Branched-chain amino acid metabolic process	GO biological process	<0.01	50.00
	GO:0022627	Cytosolic small ribosomal subunit	GO cellular component	<0.01	45.10
	GO:0006613	Cotranslational protein targeting to membrane	GO biological process	<0.01	43.81
	GO:0006614	SRP-dependent cotranslational protein targeting to membrane	GO biological process	<0.01	43.00
	GO:0045047	Protein targeting to ER	GO biological process	<0.01	42.20
	GO:0072599	Establishment of protein localization to endoplasmic reticulum	GO biological process	<0.01	41.59
	GO:0022626	Cytosolic ribosome	GO cellular component	<0.01	40.98
	GO:0006120	Mitochondrial electron transport, NADH to ubiquinone	GO biological process	<0.01	38.78
	GO:0070972	Protein localization to endoplasmic reticulum	GO biological process	<0.01	37.78
KICH					
	GO:0034497	Protein localization to phagophore assembly site	GO biological process	<0.01	58.33
	GO:0032266	Phosphatidylinositol-3-phosphate binding	GO molecular function	<0.01	40.00
	GO:0043001	Golgi to plasma membrane protein transport	GO biological process	<0.01	39.39
	GO:0080025	Phosphatidylinositol-3,5-bisphosphate binding	GO molecular function	<0.01	37.04
	KEGG:04136	Autophagy	KEGG 20.11.2017	<0.01	34.38
	GO:0007031	Peroxisome organization	GO biological process	<0.01	34.29
	GO:0061951	Establishment of protein localization to plasma membrane	GO biological process	<0.01	33.33
	GO:0015949	Nucleobase-containing small molecule interconversion	GO biological process	<0.01	32.35
	GO:0036002	Pre-mRNA binding	GO molecular function	<0.01	31.58
	GO:0006893	Golgi to plasma membrane transport	GO biological process	<0.01	30.00
KIRP					
	GO:0050136	NADH dehydrogenase (quinone) activity	GO molecular function	<0.01	41.03
	GO:0008137	NADH dehydrogenase (ubiquinone) activity	GO molecular function	<0.01	41.03
	GO:0030964	NADH dehydrogenase complex	GO cellular component	<0.01	40.00
	GO:0045271	Respiratory chain complex I	GO cellular component	<0.01	40.00
	GO:0005747	Mitochondrial respiratory chain complex I	GO cellular component	<0.01	40.00
	GO:0097031	Mitochondrial respiratory chain complex I biogenesis	GO biological process	<0.01	38.33
	GO:0010257	NADH dehydrogenase complex assembly	GO biological process	<0.01	38.33
	GO:0032981	Mitochondrial respiratory chain complex I assembly	GO biological process	<0.01	38.33
	GO:0003954	NADH dehydrogenase activity	GO molecular function	<0.01	38.10
	GO:0005753	Mitochondrial proton-transporting ATP synthase complex	GO cellular component	<0.01	38.10

**Table 4:** The information of GO terms with the most genes.

	**ID**	**Term**	**Source**	***P* value**	**Count**
KIRC					
	GO:0005622	Intracellular	GO cellular component	<0.01	1676
	GO:0044424	Intracellular part	GO cellular component	<0.01	1662
	GO:0043229	Intracellular organelle	GO cellular component	<0.01	1529
	GO:0005737	Cytoplasm	GO cellular component	<0.01	1400
	GO:0043231	Intracellular membrane-bounded organelle	GO cellular component	<0.01	1386
	GO:0044444	Cytoplasmic part	GO cellular component	<0.01	1223
	GO:0044446	Intracellular organelle part	GO cellular component	<0.01	1201
	GO:0043170	Macromolecule metabolic process	GO biological process	<0.01	1104
	GO:0044260	Cellular macromolecule metabolic process	GO biological process	<0.01	1046
	GO:0005634	Nucleus	GO cellular component	<0.01	948
KICH					
	GO:0005622	Intracellular	GO cellular component	<0.01	1271
	GO:0044424	Intracellular part	GO cellular component	<0.01	1260
	GO:0043229	Intracellular organelle	GO cellular component	<0.01	1156
	GO:0005737	Cytoplasm	GO cellular component	<0.01	1066
	GO:0043231	Intracellular membrane-bounded organelle	GO cellular component	<0.01	1050
	GO:0044444	Cytoplasmic part	GO cellular component	<0.01	959
	GO:0044446	Intracellular organelle part	GO cellular component	<0.01	920
	GO:0043170	Macromolecule metabolic process	GO biological process	<0.01	806
	GO:0044260	Cellular macromolecule metabolic process	GO biological process	<0.01	773
	GO:0005634	Nucleus	GO cellular component	<0.01	693
KIRP					
	GO:0005622	Intracellular	GO cellular component	<0.01	1064
	GO:0044424	Intracellular part	GO cellular component	<0.01	1057
	GO:0043229	Intracellular organelle	GO cellular component	<0.01	962
	GO:0005737	Cytoplasm	GO cellular component	<0.01	913
	GO:0043231	Intracellular membrane-bounded organelle	GO cellular component	<0.01	864
	GO:0044444	Cytoplasmic part	GO cellular component	<0.01	808
	GO:0044446	Intracellular organelle part	GO cellular component	<0.01	772
	GO:0044260	Cellular macromolecule metabolic process	GO biological process	<0.01	631
	GO:0005634	Nucleus	GO cellular component	<0.01	565
	GO:1901564	Organonitrogen compound metabolic process	GO biological process	<0.01	560

### Diagnostic Test

The PSI values and weighted PSI values of the 10 most significant genes and the predicted probability of the binary logistic regression equation were used to diagnose pan-RCC through the ROC curve (Figures 5D–F). The consequence indicates that not all SRGs can effectively diagnose pan-RCC. Although with the significant consequence, partial SRGs cannot be considered to have diagnostic potential, such as FAM72A_AT_6 in KIRC, DEPDC5_AT_46 in KICH, and AUH_AT_11 in KIRP. The weighted PSI values are not always predictive of pan-RCC, while the predicted probability obtained good diagnostic efficacy in each type of pan-RCC, similar to 5 year survival model (Table 5). In KIRC, there was a significant difference in all AS types. Only ADs, ATs, and RIs had significant differences in KICH. As for KIRP, significant differences were observed in all AS types except MEs (Table 6). The ROC curve plays a role in determining the predictive power of each AS type (Figures 5G–I). The AS types with AUC value >0.7 are AA, AD, AP, AT, ES, RI in KIRC, AD, AT, RI in KICH, and AD, AP, RI in KIRP. The AUC values of predicted probability were 0.935, 0.938, and 0.875 in KIRC, KICH, and KIRP, respectively, which were more reliable than the prediction by any AS type (Table 7). AD, AT, and RI had excellent performance in all subtypes.

**Table 5:** ROC curve results of survival related factors in predicting diagnostic test.

**Cancer type**	**Events**	**Cut-off**	**Sensitivity**	**Specificity**	**AUC**	**95% CI lower**	**95% CI upper**	***P*-value**
KIRC									
	Top10_KIRC	−55.416	0.827	0.806	0.887	0.851	0.923	<0.05
	C4orf19_AT_5_AT_51R	0.855	0.745	0.917	0.881	0.849	0.913	<0.05
	EPC2_AT_15	0.915	0.752	0.389	0.552	0.477	0.628	0.150
	SCP2_ES_12	0.231	0.906	0.875	0.939	0.912	0.966	<0.05
	FAM120C_AT_17	0.756	0.767	0.903	0.892	0.862	0.921	<0.05
	PCMTD1_AT_8	0.982	0.771	0.833	0.862	0.826	0.898	<0.05
	ZNF814_AT_4.2	0.129	0.795	0.861	0.883	0.848	0.919	<0.05
	INPP4B_AT_35	0.957	0.368	0.819	0.549	0.487	0.611	0.175
	FAM72A_AT_6	0.361	0.674	0.611	0.655	0.585	0.725	<0.05
	HAGH_ES_6	0.986	0.523	0.708	0.605	0.545	0.665	<0.05
	TAF1D_RI_12.4	0.238	0.402	0.889	0.629	0.577	0.681	<0.05
	Predicted probability	0.834	0.955	0.931	0.988	0.978	0.998	<0.05
KICH									
	Top10_KICH	−247.040	0.455	0.880	0.688	0.576	0.800	0.006
	TATDN1_AD_4.2	0.188	0.636	0.840	0.765	0.668	0.861	<0.05
	FAM195A_ES_3	0.655	0.409	0.760	0.552	0.421	0.683	0.444
	PLEKHB2_AD_8.2	0.142	0.682	0.520	0.557	0.428	0.686	0.401
	TATDN1_ES_3	0.938	0.470	0.760	0.603	0.476	0.730	0.131
	DPM3_AP_1	0.947	0.955	0.320	0.598	0.462	0.735	0.149
	PEX16_ES_4	0.020	0.576	0.560	0.535	0.404	0.666	0.606
	DEPDC5_AT_46	0.985	0.727	0.640	0.656	0.524	0.788	<0.05
	BCL2L13_ES_7	0.892	0.879	0.920	0.940	0.891	0.989	<0.05
	MRPS24_RI_1.2	0.097	0.561	0.880	0.683	0.578	0.789	<0.05
	UBAP2L_AT_29	0.382	0.364	0.920	0.593	0.479	0.707	0.174
	Predicted probability	0.614	0.955	0.920	0.970	0.936	1.000	<0.05
KIRP									
	Top10_KIRP	−375.286	0.341	0.906	0.502	0.425	0.571	0.973
	COPE_ES_4	0.996	0.562	0.844	0.735	0.651	0.819	<0.05
	PPP1CA_ES_2.2	0.989	0.614	0.813	0.697	0.622	0.772	<0.05
	RBM39_AT_24	0.983	0.648	0.844	0.737	0.664	0.809	<0.05
	PKIG_ES_2.2	0.187	0.531	0.906	0.701	0.627	0.776	<0.05
	CLDN11_AT_3	0.261	0.779	1.000	0.918	0.886	0.951	<0.05
	FKBP8_AA_6.1	0.723	0.779	0.844	0.839	0.774	0.904	<0.05
	GLS_AT_20	0.873	0.859	0.531	0.723	0.622	0.824	<0.05
	GUK1_AA_7.1	0.964	0.431	0.719	0.534	0.443	0.625	0.527
	KIF4A_AT_32	0.210	0.741	0.813	0.829	0.763	0.895	<0.05
	AUH_AT_11	0.890	0.334	0.969	0.618	0.539	0.696	<0.05
	Predicted probability	0.923	0.979	1.000	0.999	0.997	1.000	<0.05

*Events are identified as symbol_splice type_exons. Top10_KIRC means the weighted PSI value of the top10 significant SREs in KIRC. Top10_KICH means the weighted PSI value of the top10 significant SREs in KICH. Top10_KIRP means the weighted PSI value of the top10 significant SREs in KIRP.AUC, area under the ROC curve; CI, confidence interval*.

**Table 6:** Difference of PSI value between pan-RCC and normal tissues in each type of alternative splicing.

**Splice events**		**KICH**			**KIRC**			**KIRP**	
	**Cancer**	**Normal**	***P***	**Cancer**	**Normal**	***P***	**Cancer**	**Normal**	***P***
AA	0.619 ± 0.007	0.618 ± 0.003	0.266	0.620 ± 0.013	0.612 ± 0.004	<0.001	0.622 ± 0.014	0.614 ± 0.006	<0.001
AD	0.546 ± 0.010	0.540 ± 0.004	<0.001	0.546 ± 0.014	0.531 ± 0.005	<0.001	0.555 ± 0.015	0.543 ± 0.007	<0.001
AP	0.831 ± 0.011	0.834 ± 0.005	0.197	0.819 ± 0.018	0.832 ± 0.004	<0.001	0.820 ± 0.020	0.832 ± 0.005	<0.001
AT	0.852 ± 0.006	0.858 ± 0.011	0.002	0.859 ± 0.016	0.869 ± 0.007	<0.001	0.858 ± 0.010	0.864 ± 0.010	<0.001
ES	0.714 ± 0.015	0.710 ± 0.003	0.729	0.718 ± 0.017	0.707 ± 0.003	<0.001	0.740 ± 0.017	0.731 ± 0.005	<0.001
ME	0.471 ± 0.010	0.469 ± 0.007	0.241	0.467 ± 0.010	0.463 ± 0.007	0.004	0.487 ± 0.012	0.487 ± 0.007	0.764
RI	0.585 ± 0.018	0.571 ± 0.009	<0.001	0.604 ± 0.034	0.570 ± 0.013	<0.001	0.609 ± 0.032	0.580 ± 0.019	<0.001

**Table 7:** ROC curve results of PSI of each AS types in diagnostic test.

**Cancer type**	**Type**	**Cut-off**	**Sensitivity**	**Specificity**	**AUC**	**95% CI lower**	**95% CI upper**	***P*-value**
KIRC								
	AA	0.616	0.578	0.917	0.715	0.673	0.758	<0.05
	AD	0.538	0.653	0.972	0.848	0.813	0.882	<0.05
	AP	0.828	0.666	0.903	0.823	0.785	0.862	<0.05
	AT	0.862	0.548	0.875	0.720	0.674	0.767	<0.05
	ES	0.709	0.707	0.806	0.796	0.755	0.838	<0.05
	ME	0.470	0.332	0.875	0.604	0.541	0.667	<0.05
	RI	0.587	0.638	0.944	0.814	0.775	0.852	<0.05
	Predicted probability	0.908	0.799	0.958	0.935	0.912	0.959	<0.05
KICH								
	AD	0.540	0.803	0.720	0.779	0.676	0.882	<0.05
	AT	0.859	0.864	0.560	0.708	0.565	0.851	<0.05
	RI	0.572	0.864	0.640	0.792	0.688	0.896	<0.05
	Predicted probability	0.775	0.833	0.960	0.938	0.888	0.988	<0.05
KIRP								
	AA	0.616	0.645	0.719	0.695	0.612	0.777	<0.05
	AD	0.545	0.790	0.688	0.793	0.716	0.869	<0.05
	AP	0.827	0.586	0.906	0.770	0.706	0.834	<0.05
	AT	0.866	0.817	0.594	0.714	0.607	0.821	<0.05
	ES	0.732	0.645	0.781	0.704	0.625	0.784	<0.05
	RI	0.598	0.597	0.875	0.787	0.714	0.859	<0.05
	Predicted probability	0.855	0.831	0.781	0.875	0.820	0.930	<0.05

*AUC, area under the ROC curve; CI, confidence interval*.

### Survival-Related SFs and Regulatory Network

**Figure 6:**
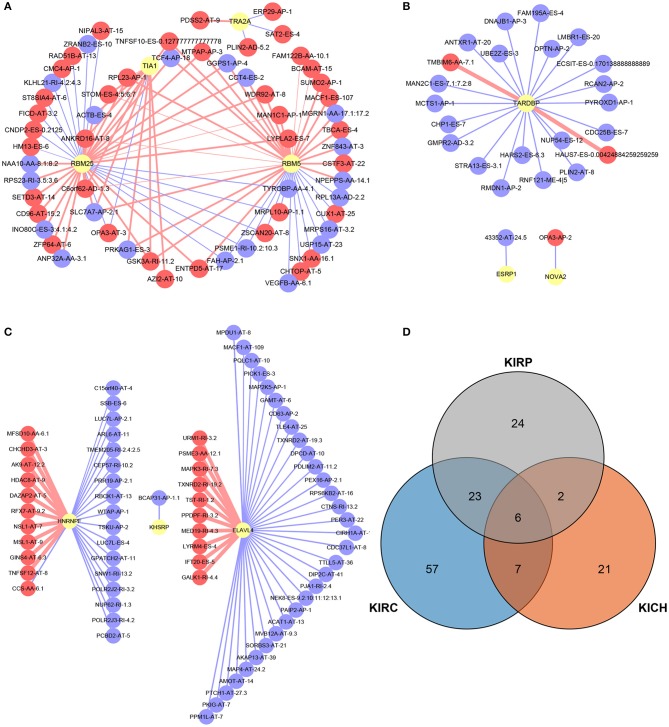
SF-AS events regulatory network and the distribution of the key genes in PPI network. **(A–C)** The SF-AS events regulatory networks in KIRC, KICH, and KIRP, respectively. The red nodes represented the AS events that were positively related to survival. The purple nodes represented the AS events that were negatively related to survival. The red and purple lines represented a positive and negative correlation between the connected nodes, respectively. The larger the correlation coefficient was, the thicker the line was and vice versa. **(D)** The distribution of the key genes in PPI network. The orange, blue, and gray areas represent the genes in KIRC, KICH, and KIRP, respectively. The genes shown in overlapping regions exist in all relevant groups.

A total of 12, 9, and 6 SFs were associated with prognosis of KIRC, KICH, and KIRP, and their effects on prognosis were marked by different colors in Figures 5J–L. At the threshold = 0.4, 4689, 966 and 226 SF-AS event pairs were found in KIRC, KICH, and KIRP, respectively, some of which with larger Pearson correlation coefficients were shown in Figures 6A–C. In KICH and KIRP, most of the SFs were negatively related to AS events that prolong survival, while such trend was not apparent in KIRC.

## Discussion

The phenomenon of AS was noticed in the twentieth century, but it has not been systematically analyzed. Advances in high-throughput sequencing technology allows us to explain the rapport between abnormal AS events and pan-cancer at the genome-wide level. Aberrant AS events have been proven to interfere with the initiation and progression of several cancers. Protein is the bearer of life activities and acts directly on regular or deviant life activities. The generation of protein diversity depends on the precisely regulated AS events that occur in pre-mRNA (28, 29). Compared to genetic mutations, AS has a broader and more direct effect on proteins. Once the AS event is out of precise regulation, deviant pre-mRNA modifications are produced and disrupt the stability of the transcriptome, becoming a potential risk factor for cancer (4). For instance, BC200 cooperates with hnRNP A2/B1 and Sam68 to regulate AS of Bcl-x-pre-mRNA in breast cancer patients. This interaction eventually inhibits Bcl-xS expression, but simultaneously up-regulates Bcl-xL expression, which promotes tumor cell proliferation and increasing resistance to anti-cancer therapies (30). Single AS event like this is only a microcosm of cancer development and progression. Further, some researchers have found that about half of all AS events in ovarian and breast tissue have abnormal changes in tumor tissue (31). Previous analyses of small-scale AS events inspire us to follow the significance of AS events for the course of pan-RCC and their potential as predictors (15, 32–35).

The current classification is based on the pathological features of the tumor, and we sought to investigate the association between various subtypes of pan-RCC through the distribution of all AS events and SREs. We piloted computational biology methods to correlate pan-RCC with large-scale AS events, and mine the characteristics of AS events occurring in pan-RCC at the genome-wide level, providing a new perspective for the diagnosis and treatment of Pan-RCC. We identified SREs from genome-wide levels in patients with KIRC, KICH, and KIRP. We found that although most of the subtypes of pan-RCC have the same AS events and ASRGs, there were significant differences between their SREs and SRGs, which might be the source of differences in subtypes. In particular, when analyzing the distribution of SREs in subtypes, we found that the SREs for KIRC and KIRP are primarily AT, while ES in KICH, suggesting that KIRC and KIRP have similarities in disease progression. KICH seems to have different molecular mechanisms. Furthermore, we analyzed the cross-subtype distribution of SREs and SRGs. Surprisingly, under the condition of confidence = 0.9, SRGs involved in the PPI network indicates that KIRP and KIRC own many identical genes, which means nearly half of the genes in the PPI network of KIRP are also present in that of KIRC (Figure 6D). Some views believe that KIRC and KIRP are two tumors with low association in pathological changes (6), prognosis (36) and imaging changes (37). In clinical practice, clear cell papillary RCC with dual features of KIRC and KIRP was discovered and suggested as an independent type of RCC (38). Functional enrichment analysis makes known that most of the SRGs in pan-RCC have the same biological function, and the heterogeneity of the tumor depends on some key genes. This seems to expound that although KIRC and KIRP have many of the same SRGs, they are considered to be two distinct tumors based on pathological features. In summary, the current study confirms that KIRC and KIRP do have common molecular characteristics through analysis of SRGs associated with AS events, and the key to figuring out the difference between the two is analyzing the functions of the relevant genes.

Furthermore, in order to identify the effects of SREs on the occurrence and prognosis of pan-RCC, we disclosed gene function and participation pathways through enrichment analysis and found that a series of single SRE had an impact on the survival of patients, indicating it has potential to be therapeutic target point. For instance, the branched-chain amino acid catabolic process enriched in KIRC is a vital biological metabolic step, closely related to cancer (39). Many studies have clarified that cancer has specific metabolic characteristics, an important direction for studying cancer (40, 41). The enrichment of KIRP is mainly related to the oxidative respiratory chain, in which inhibition of NADH dehydrogenase activity has been proven to promote gastric cancer and breast cancer (42, 43). Further research should focus on the existence of similar mechanisms in KIRP. Survival curves with SREs as molecular features displayed that AS events had significant impact on patients' survival. In particular, if we combined multiple events, a larger difference would be detected between the two groups. Multiple studies have used SREs as molecular features for the diagnosis and prognosis of cancer (44). Unfortunately, previous studies have always analyzed prognostic-related factors independently by individual or category. Multi-factor models often exhibit better consequences than single-factor models in the diagnosis and prediction of prognosis. When building a multi-factor model, we selected a binary logistic regression equation instead of a weighted PSI value and obtained a better performance in this study. Multivariate analysis established a univariate predictive model to compare their effects. In the 5 year survival model, multivariate prediction illustrated better accuracy than univariate ones. Diagnostic tests had also provided similar results, emphasizing that when AS events are used as predictors of disease, they should be integrated rather than by individual or type. The binary logistic regression equation demonstrated superior performance in all analyses and was accepted as an excellent model for diagnosing pan-RCC and evaluating patient prognosis. Furthermore, in order to figure out the pathological and physiological mechanisms of AS events, we constructed an SF-AS event regulatory network. Recent studies have revealed that SFs are closely related to the tumorgenesis and can serve as potential therapeutic targets (45). Some researchers have noted this phenomenon and studied AS events in hepatocellular carcinoma, lung cell carcinoma and RCC (46–48). While our research points out the direction for subsequent research by mining survival-related SFs and constructing regulatory networks for SF-AS events. Surprisingly, some SFs are negatively related to AS events that reduce survival, whereas SF itself is negatively related to survival, suggesting that the relevant AS event is not the only way that the SFs affects the prognosis of the disease. The role of AS events in pan-RCC is complex and comprehensive, and more details deserve to be studied.

Despite the findings, some limitations should be addressed. For instance, SREs used to fit binary logistic regression equations need to be further extracted from all SREs, which can increase the representativeness of the variables and the stability of the equations. All consequences should be tested in another set of samples to determine the reliability of the results as well. More specific mechanisms of AS affecting pan-RCC should be dig deeper to find available therapeutic targets.

Collectively, our study systematically analyzed transcriptome-wide AS events and identified novel SREs among KIRC, KICH, and KIRP, thus providing the foundation for subsequent research on therapeutic targets.

## Data Availability Statement

The datasets analyzed for this study can be found in TCGA [https://portal.gdc.cancer.gov/], TCGA SpliceSeq [https://bioinformatics.mdanderson.org/public-software/tcgaspliceseq/], and SpliceAid 2 [www.introni.it/spliceaid.html].

## Author Contributions

HW, KJ, and YW contributed conception and design of the study. KJ organized the database and wrote the first draft of the manuscript. KJ and YW performed the statistical analysis. HW, YW, and JH wrote sections of the manuscript. All authors contributed to manuscript revision, read, and approved the submitted version.

### Conflict of Interest

The authors declare that the research was conducted in the absence of any commercial or financial relationships that could be construed as a potential conflict of interest.
