# Supplementary material for: Survival-Associated Alternative Splicing Events in Pan-Renal Cell Carcinoma
Source: Front Oncol. 2019 Nov 27;9:1317. doi: 10.3389/fonc.2019.01317 (PMC6902018; doi:10.3389/fonc.2019.01317)
Supplement: Supplementary file 2 [file Data_Sheet_1.docx]

**Supplementary Figure 1.** The PPI network of SREs.

The more nodes that are connected, the more the nodes tend to be red; the other way they tend to be white. The larger the co-expression coefficient of the nodes connected by lines, the thicker the lines, and the thinner the opposite. (a)renal clear cell carcinoma; (b)renal chromophobe cell carcinoma; (c)renal papillary cell carcinoma
