# Supplementary figures and images for: Survival-Associated Alternative Splicing Events in Pan-Renal Cell Carcinoma
Source: Front Oncol. 2019 Nov 27;9:1317. doi: 10.3389/fonc.2019.01317 (PMC6902018; doi:10.3389/fonc.2019.01317)

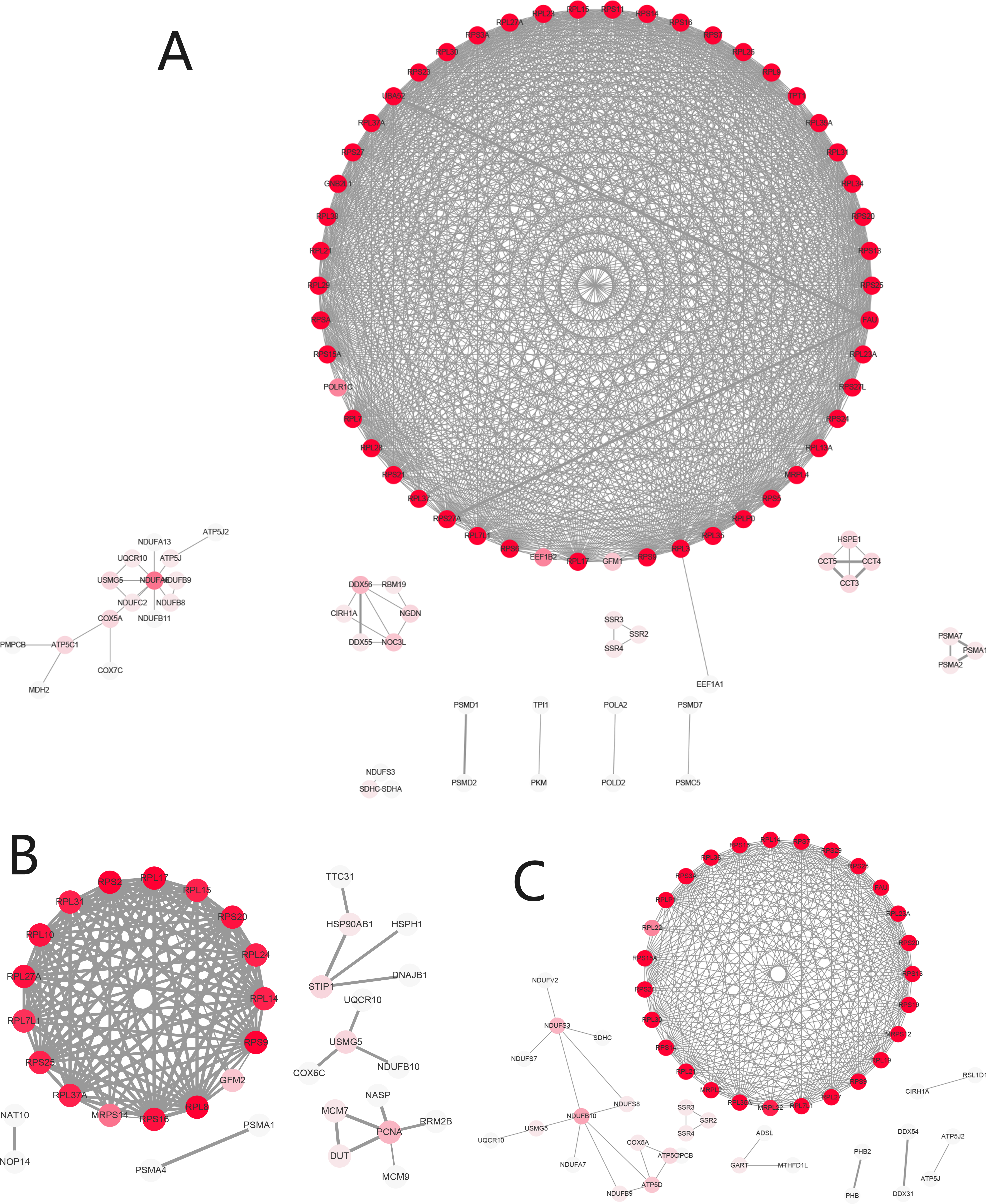

Supplement: Supplementary file 3 [file Image_1.TIF]
